# A novel autophagy activator ginsenoside Rh2 enhances the efficacy of immunogenic chemotherapy

**DOI:** 10.1002/ctm2.1109

**Published:** 2023-02-02

**Authors:** Jing Yang, Wei Zhang, Lin Jia, Fei Shi, Min Cao, Jichao Sun, Chengchao Xu, Zhijie Li, Zhiqiang Cheng, Shan‐Chao Zhao, Jigang Wang, Chuanbin Yang

**Affiliations:** ^1^ Department of Nephrology Shenzhen People's Hospital (The Second Clinical Medical College, Jinan University, The First Affiliated Hospital, Southern University of Science and Technology) Shenzhen China; ^2^ College of Pharmacy Shenzhen Technology University Shenzhen China; ^3^ Emergency Department, Institute of Shenzhen Respiratory Diseases Shenzhen People's Hospital (The Second Clinical Medical College, Jinan University, The First Affiliated Hospital, Southern University of Science and Technology) Shenzhen China; ^4^ Department of Pathology Shenzhen People's Hospital (The Second Clinical Medical College, Jinan University, The First Affiliated Hospital, Southern University of Science and Technology) Shenzhen China; ^5^ Department of Urology The Third Affiliated Hospital of Southern Medical University Guangzhou China; ^6^ Department of Urology Nanfang Hospital, Southern Medical University Guangzhou China; ^7^ Artemisinin Research Center, Institute of Chinese Materia Medica China Academy of Chinese Medical Sciences Beijing China

Dear Editor,

Immunogenic cell death (ICD) caused by certain chemotherapeutic drugs, including mitoxantrone (MTX), elicits specific protective anti‐tumour immunity and is, thus, regarded as an effective strategy for cancer treatment. Pharmacological enhancement of autophagy is effective in enhancing anticancer immune responses to ICD‐inducing chemotherapeutic drugs. Here, we discover that ginsenoside Rh2 (G‐Rh2) enhance MTX‐induced hallmarks of ICD, which include increased ATP release, relocation of calreticulin (CALR) to the cell membrane and HMGB1 (high mobility group box 1) secretion. Mechanistic studies reveal that G‐Rh2induces autophagy through the activation of TFEB (transcription factor EB) and TFE3 (transcription factor E3), which contributes to the synergistic effect of G‐Rh2 and MTX on promoting ATP release. In addition, G‐Rh2 increased endoplasmic reticulum (ER) stress with phosphorylated eukaryotic initiation factor eIF2α, which promoted MTX‐induced cell surface calcineurin exposure. Consequently, G‐Rh2 enhanced the in vivo anti‐tumour effect of MTX in immunocompetent mice bearing MCA205 tumour with increased cytotoxic T lymphocytes (CTLs). Thus, G‐Rh2 represents a promising drug candidate for treating cancers in combination with ICD‐inducing chemoimmunotherapeutic drugs such as MTX.

In response to certain cellular stimuli, injured or stressed cells release DAMPs on their surface to produce immunostimulatory effects, including recruiting and activating immune cells that ultimately kill cancer cells.^1^ This kind of regulated cell death is referred to as ICD.^1^, ^2^ ICD can be triggered by multiple chemotherapeutics such as oxaliplatin and MTX. Key hallmarks of ICD include the secretion of ATP, cell surface relocation of CALR and extracellular release of HMGB1.^2^ Extracellular ATP acts as a ‘find me’ molecule that recruits antigen‐presenting cells to promote anticancer immunity. Cell membrane CALR acts as an ‘eat me’ molecule for dendritic cells (DCs) to capture antigens and trigger tumour‐specific cytotoxic T‐cell responses. Extracellular HMGB1 binds to its receptor such as TLR4 on DCs, which promotes tumour antigen processing and presentation to T cells. Thus, the induction of ICD triggers long‐lasting anti‐tumour immunity, and it is regarded as an effective strategy for cancer treatment.^2^, ^3^

TFEB and TFE3 are key transcription factors that regulate autophagy.^4^, ^5^ With respect to ICD, the activation of several stress pathways, including autophagy, is indispensable for intracellular ATP release.^6^ Induction of autophagy by several ICD inducers enhances the anticancer effects via modulating the tumour microenvironment.^7^ Therefore, autophagy activation to enhance the effects of chemotherapeutics on inducing ICD holds promise for anticancer therapy.^8^ Driven by these considerations, we sought to identify novel autophagy enhancer(s) and evaluate their roles in stimulating anticancer immunity in combination with ICD‐inducing chemotherapeutics in U2OS cells (human bone osteosarcoma epithelial cells), MCA205 cells (mouse fibrosarcoma cells) and MCA205‐inoculated immunocompetent mice.

Here, we found that G‐Rh2 upregulated the autophagy marker LC3‐II levels (Figure 1A), and lysosomal inhibitor CQ further enhanced LC3‐II levels (Figure 1B,C). Immunostaining results further showed that G‐Rh2 increased autophagosomes and autolysosomes (Figure 1D–G). These results indicate that G‐Rh2 promotes autophagy. Furthermore, G‐Rh2 enhanced the nuclear accumulation of TFEB and TFE3 as reflected by immunofluorescence (Figures 1H,I and S1A,C) and western blotting (Figure S1B,D). Knock‐down of the expression of both TFEB and TFE3 (Figure S1E–H, Table S1) inhibited G‐Rh2‐induced autophagic flux (Figure S1I). Furthermore, G‐Rh2 promoted TFEB dephosphorylation (Figure S1J), and the nuclear accumulation of TFEB/TFE3 is earlier than autophagy induction (Figure S1K,L). These results suggest that G‐Rh2 enhances autophagy via TFE3 and TFEB.

**FIGURE 1:**
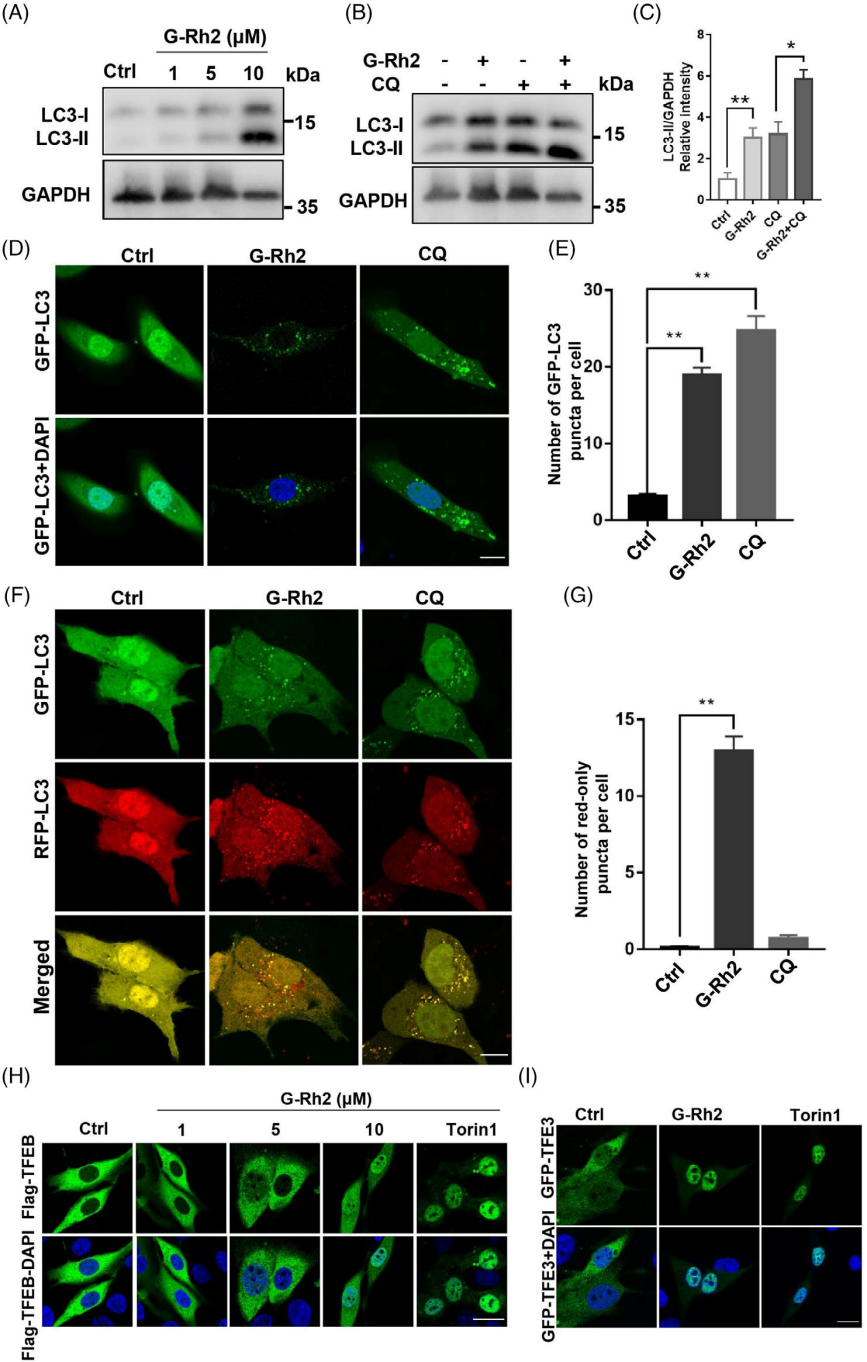
Ginsenoside Rh2 (G‐Rh2) induces autophagy via transcription factor EB (TFEB) and transcription factor E3 (TFE3) activation: (A) G‐Rh2 increases LC3‐II levels. U2OS cells were exposed to different doses of G‐Rh2 (1, 5 and 10 μM) for 16 h, and LC3‐II was measured; (B and C) G‐Rh2 induces autophagic flux. U2OS cells were exposed to G‐Rh2 (10 μM) with or without CQ (50 μM, added at last for 3 h) for 16 h, LC3‐II was measured (B) and quantified by ImageJ (C); (D and E) G‐Rh2 increases LC3 puncta. After treating U2OS cells transiently expressing GFP‐LC3 with G‐Rh2 for 16 h, LC3 puncta was visualized (D) and quantified (E); (F and G) G‐Rh2 increases autolysosomes. After treating U2OS cells transiently expressing GFP‐RFP‐LC3 with G‐Rh2 (10 μM) for 16 h, LC3 puncta was recorded (F) and red‐only puncta (autolysosome) was quantified (G). Scale bar: 15 μm; (H and I) G‐Rh2 induces the relocation of TFEB and TFE3 from the cytoplasm into the nucleus. U2OS cells transiently expressing 3XFlag‐TFEB or GFP‐N1‐TFE3 were incubated with indicated doses of G‐Rh2 (1, 5 and 10 μM) for 16 h. The distribution of TFEB in cells was detected by fluorescence microscope. Scale bar: 15 μm. *p < .05; **p < .01

We further determined whether G‐Rh2 induces hallmarks of ICD with or without a low concentration of MTX (MTX_low_). G‐Rh2 or MTX slightly but significantly reduced intracellular ATP release, and G‐Rh2 combined with MTX_low_ substantially reduced the intracellular ATP contents (Figure 2A,B). Autophagy deficiency by knocking down *ATG5* (Figure 2C–E) attenuated G‐Rh2 plus MTX_low_‐induced decrease in intracellular ATP contents as reflected by quinacrine staining (Figure 2F)^9^ and the release of extracellular ATP contents (Figure 2G). Similarly, the combination of G‐Rh2 and MTX‐induced decrease of intracellular ATP and increase of extracellular ATP was inhibited in *TFE3*‐ and *TFEB*‐knocked‐down cells (Figure 2H,I). These findings demonstrate that the synergistic effect of G‐Rh2 and MTX on ATP release depends on autophagy induction.

**FIGURE 2:**
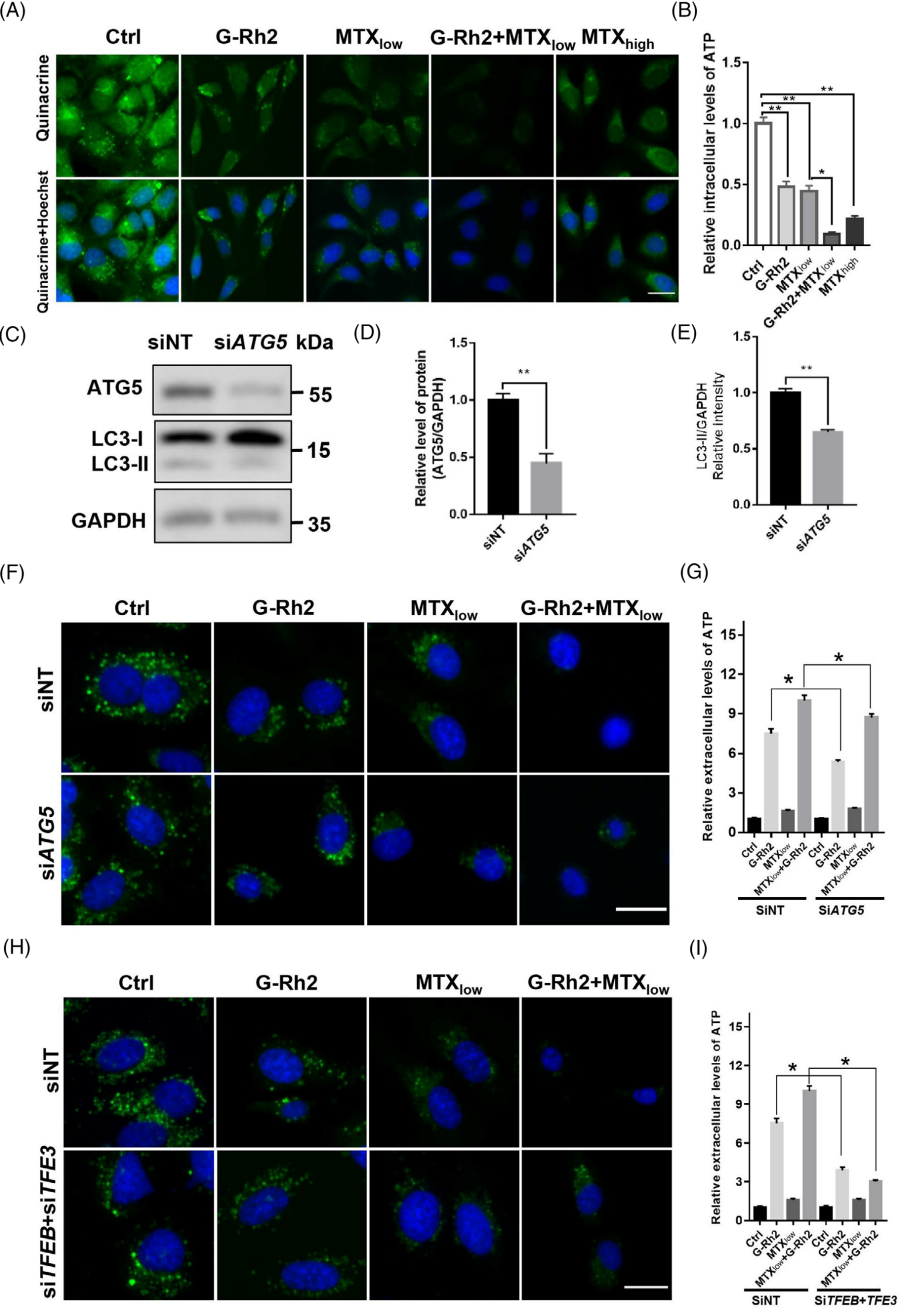
Ginsenoside Rh2 (G‐Rh2) enhances autophagy‐dependent ATP release: (A) G‐Rh2 reduces intracellular ATP contents. U2OS cells were exposed to vehicle control, G‐Rh2 (10 μM), a low dose of MTX_low_ (1 μM) or their combination for 16 h. The intracellular ATP contents were examined by quinacrine staining. MTX_high_ (5 μM) was used as a positive control. Scale bar: 15 μm; (B) quantification data in (A) shows that G‐Rh2 promotes mitoxantrone (MTX)‐induced reduction of intracellular ATP contents; (C–E) after transfected U2OS cells with siRNA to knock down the expression of key autophagy gene *ATG5*, ATG5 and LC3‐II levels were measured (C) and quantified (D and E); (F and G) the inhibition of autophagy through knocking down of the expression of ATG5 attenuates G‐Rh2 plus MTX‐induced ATP release. After *ATG5* knocking down, U2OS cells were treated with vehicle control, G‐Rh2 (10 μM), MTX_low_ (1 μM) or the combination of G‐Rh2 (10 μM) and MTX_low_ (1 μM) for 16 h, the intracellular ATP levels were measured by quinacrine staining (F) and the extracellular ATP contents were measured by a bioluminescent assay kit (G); (H and I) inhibition of autophagy by transcription factor EB (TFEB)/transcription factor E3 (TFE3) knockdown attenuates G‐Rh2 plus MTX‐induced ATP release. After *TFE3* and *TFEB* knockdown, U2OS cells were incubated with G‐Rh2, MTX_low_, or the combination of G‐Rh2 (10 μM) and MTX_low_ (1 μM) for 16 h, and the intracellular ATP contents were measured by quinacrine staining (H), and the extracellular ATP contents were measured by a bioluminescent assay kit (I). Scale bar: 15 μm. *p < .05, **p < .01

Furthermore, G‐Rh2 increased MTX_low_‐induced cell surface exposure of CALR as reflected by immunostaining and flow cytometry analysis (Figures 3A–C and S2A,B). The combination of G‐Rh2 and MTX_low_ also increased an HMGB1 release (Figure 3D–G). To determine how G‐Rh2 and MTX induce cell surface CALR exposure, we next showed that G‐Rh2 increased ER stress, especially PERK/p‐eIF2α/ATF4 axis (Figure S3A–H). We discovered that PERK knock‐down reduced G‐Rh2‐induced ER stress (Figure S3I–K) and comprised G‐Rh2 plus MTX‐caused cell surface CALR exposure (Figure 3H,I). Interestingly, the inhibition of ER stress by 4‐PBA (4‐phenylbutyric acid) also attenuated cell surface relocation of CALR (Figure S3L). These results indicate that ER stress is indispensable for the role of G‐Rh2 in enhancing MTX‐induced cell surface relocation of CALR. Apart from ICD, the combination of G‐Rh2 and MTX also induced cell apoptosis, and this effect was further enhanced by the lysosomal inhibitor CQ (Figure S4A,B), suggesting that the apoptosis may also be involved in anticancer effects. To understand the crosstalk of autophagy and ER stress during ICD, we found that the inhibition of lysosomal functions by CQ did not further enhance ER stress (Figure S4C), and ER stress inhibitor 4‐PBA attenuated autophagy in response to G‐Rh2 (Figure S4D). Consistently, CQ did not enhance G‐Rh2 plus MTX‐induced cell surface CALR exposure (Figure S4E) but attenuated G‐Rh2 plus MTX‐induced ATP release (Figure S4F), supporting a critical role of autophagy in promoting ATP release. Furthermore, though apoptosis inhibitor Z‐VAD‐FMK inhibits G‐Rh2 plus MTX‐induced apoptosis (Figure S5B), Z‐VAD‐FMK did not inhibit G‐Rh2 plus reduction of intracellular ATP levels (Figure S5A), and cell surface CALR exposure (Figure S5C,D), further strengthen the hypothesis that ICD rather than apoptosis is involved in the anti‐tumour effect of G‐Rh2 plus MTX.

**FIGURE 3:**
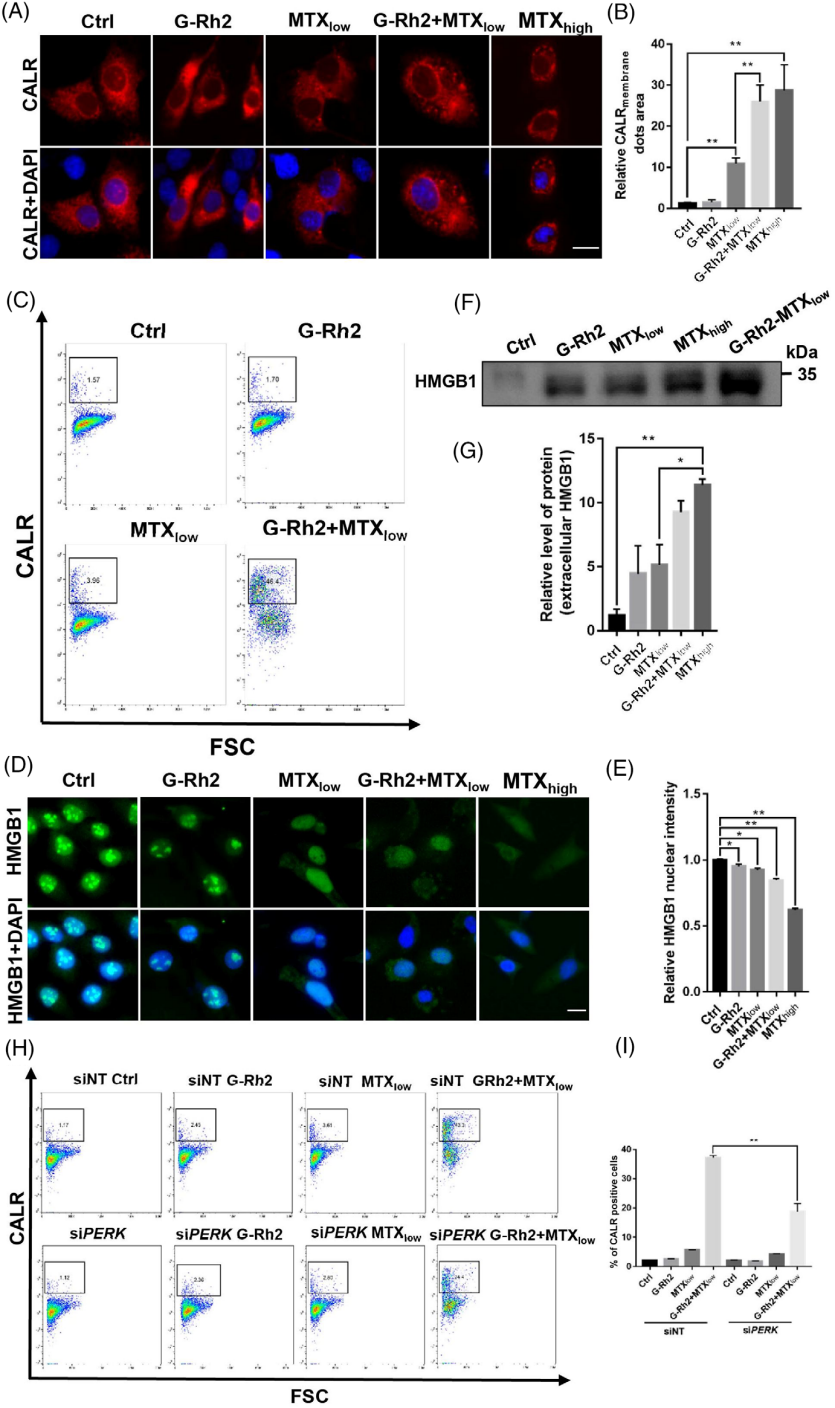
Ginsenoside Rh2 (G‐Rh2) enhances the cell surface exposure of calreticulin (CALR) and the release of HMGB1 (high mobility group box 1) in the presence of mitoxantrone (MTX): (A) G‐Rh2 enhances MTX‐induced cell surface relocation of CALR. U2OS cells transiently expressing CALR‐KDEL‐RFP were treated with a low concentration of MTX_low_ (1 μM) with or without G‐Rh2 (10 μM) for 16 h. The cell surface CALR was visualized by a confocal microscope. MTX_high_ (5 μM) was used as a positive control. Scale bar: 15 μm; (B) quantification of cell surface CALR exposure in (A); (C) G‐Rh2 enhances MTX‐induced cell surface CALR exposure measured by flow cytometry. After drug treatment as shown in (A), U2OS cells were collected for the detection of endogenous CALR exposure via flow cytometry; (D–G) G‐Rh2 enhances MTX‐induced HMGB1 release. Intracellular HMGB1 was detected by immunostaining after drug treatment for 24 h (D) and quantified (E). Extracellular HMGB1 contents in cell culture medium were detected by western blotting after drug treatment (F) and quantified (G). Briefly, an equal amount of cell culture medium was precipitated using trichloroacetic acid followed by western blotting analysis. Scale bar: 15 μm; (H and I) inhibition of endoplasmic reticulum (ER) stress by knocking down of PERK, a key molecule in ER stress, attenuates G‐Rh2‐induced cell surface CALR exposure as determined by flow cytometry. *p < .05; **p < .01

To confirm the conserved synergistic effects of G‐Rh2  and MTX in enhancing ICD, we showed that in immunosurveillance MCA205 mouse fibrosarcoma cells, G‐Rh2 also enhanced autophagy (Figure S6A), induced ER stress (Figure S6B,C). Consistently, G‐Rh2 enhanced MTX_low_‐induced cell surface CALR exposure, HMGB1 release from the nucleus, and extracellular ATP release (Figure S6D–I), suggesting that G‐Rh2 also promotes MTX‐induced ICD in MCA205 fibrosarcoma cells. MCA205 cells inoculated in mice are well characterized as a suitable model for the investigation of immune response, and the tumour infiltration on the skin can also be considered to be orthotopic.^10^ We next determined the synergistic anti‐tumour role of G‐Rh2 in combination with MTX by inoculating MCA205 cells into immunocompetent C57 mice followed by drug treatment as shown in the schematic model (Figure 4A). We showed that G‐Rh2, MTX and a combination of G‐Rh2 and MTX did not affect mice's body weight (Figure 4B), but the combination treatment significantly mitigated tumour growth (Figure 4C,D). Importantly, the combination treatment increased the abundance of CTLs while exerting minimal effect on that of regulatory T cells (Tregs) (Figures 4E,F and S7). Consequently, this combination treatment increased the CTL/Treg ratio (Figure 4G), suggesting that G‐Rh2 and MTX synergistically promote anti‐tumour immunity by tipping the immune balance and reprogramming the tumour microenvironment.

**FIGURE 4:**
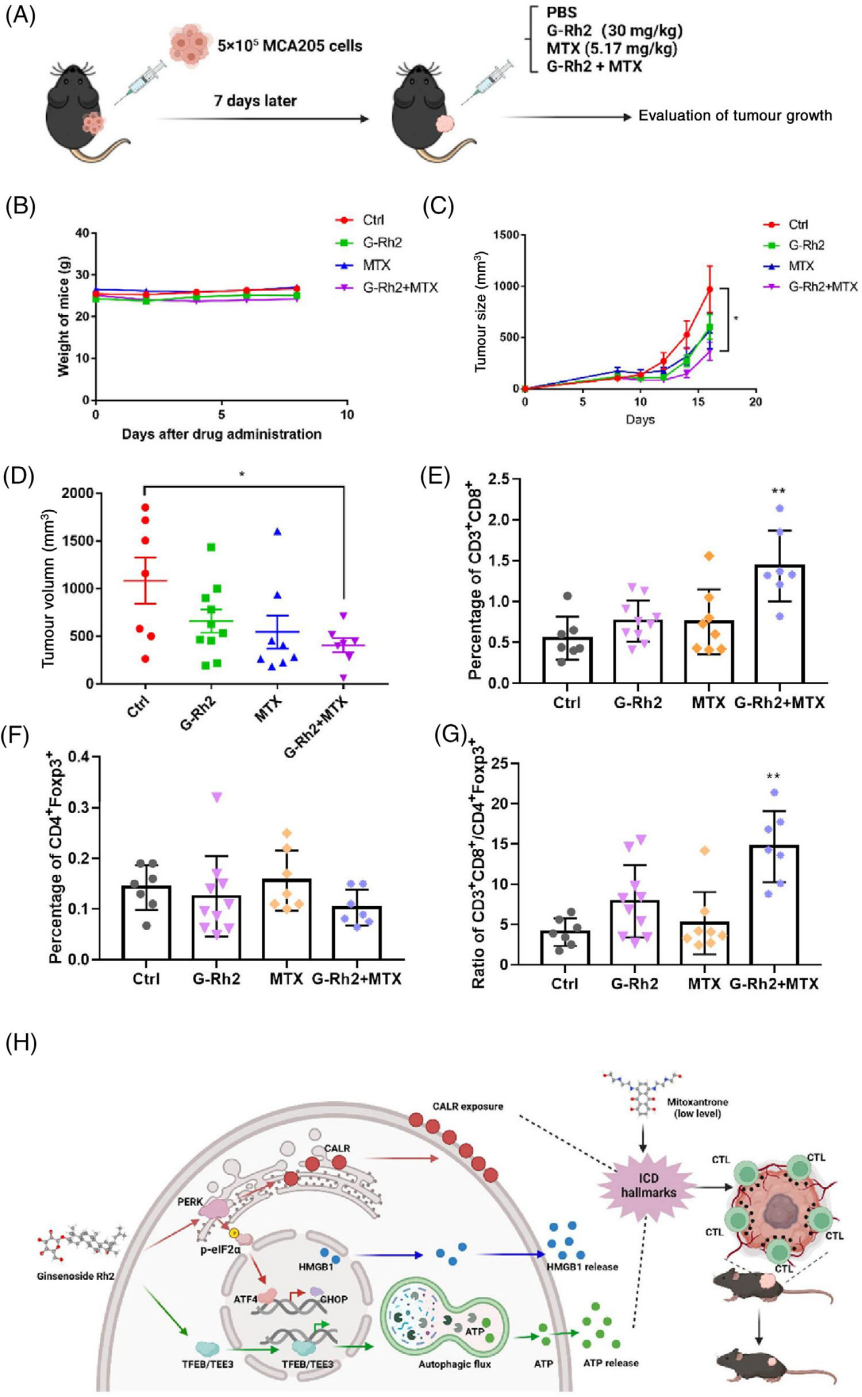
Ginsenoside Rh2 (G‐Rh2) promotes the efficacy of anticancer chemotherapy in mice: (A) schematic diagram of drug treatment in MCA205 mouse fibrosarcomas‐bearing mice. When tumours became palpable, mice received systemic intraperitoneal injection of G‐Rh2, mitoxantrone (MTX) alone or their combination. At least seven mice per group; (B) treatment of mice with G‐Rh2, MTX alone or their combination does not affect mice's body weight; (C and D) G‐Rh2 plus MTX treatment significantly inhibits tumour growth (C) and reduces tumour size (D) of MCA205 fibrosarcomas in mice; (E) G‐Rh2 plus MTX increases the ratio of CD3^+^CD8^+^ cytotoxic T lymphocytes (CTLs); (F) treatment of mice with G‐Rh2, MTX alone or in combination does not affect the ratio of CD4^+^ FOXP3^+^ Treg cells; (G) treatment of mice with the combination of G‐Rh2 and MTX increases the ratio of CD3^+^ CD8^+^ T lymphocytes over Treg cells; *p < .05; **p < .01. (H) a schematic model illustrating the effects of G‐Rh2 in enhancing MTX‐induced immunogenic cell death (ICD) and promoting its anti‐tumour effects via reprogramming the tumour microenvironment. G‐Rh2 enhances MTX‐induced hallmarks of ICD, such as ATP release, cell surface calreticulin (CALR) exposure and HMGB1 (high mobility group box 1) release. Mechanistically, G‐Rh2 promotes MTX‐induced ATP release via transcription factor EB (TFEB)/transcription factor E3 (TFE3)‐mediated autophagy, and G‐Rh2 facilitates MTX‐induced cell surface CALR exposure via activating endoplasmic reticulum (ER) stress through PERK/p‐eIF2α/ATF4 axis. As such, G‐Rh2 synergizes with MTX to increase the abundance of CTLs in tumours, which ultimately promotes the in vivo anti‐tumour effects of chemotherapeutic ICD‐inducer MTX.

Overall, this study illustrates that G‐Rh2 is responsible for TFE3/TFEB‐mediated autophagy activation and ER‐stress induction with phosphorylated eIF2α, and it synergizes with immunogenic chemotherapeutic drug MTX to enhance MTX‐induced ICD, which consequently facilitates the anti‐tumour effect of MTX in immunocompetent mice in vivo (Figure 4G). Our findings provide mechanistic insights into how G‐Rh2 synergizes with MTX to amplify its effects on ICD induction and anti‐tumour activity and provide a novel link between G‐Rh2‐activated TFEB/TFE3‐dependent autophagy induction and ICD‐involved anti‐tumour effect. Our discovery indicates that G‐Rh2 is a novel drug candidate for improving the anti‐tumour effects of immunogenic chemotherapies.

## CONFLICTS OF INTEREST

There are no conflicts of interest between all authors.

## Supporting information

Figures

Supporting Information

## Data Availability

Data are available on reasonable request from the authors.
