# Supplementary material for: A novel autophagy activator ginsenoside Rh2 enhances the efficacy of immunogenic chemotherapy
Source: Clin Transl Med. 2023 Feb 2;13(2):e1109. doi: 10.1002/ctm2.1109 (PMC9894730; doi:10.1002/ctm2.1109)
Supplement: Supplementary file 1 — Figures [file CTM2-13-e1109-s002.docx]

**Supplementary Figures**


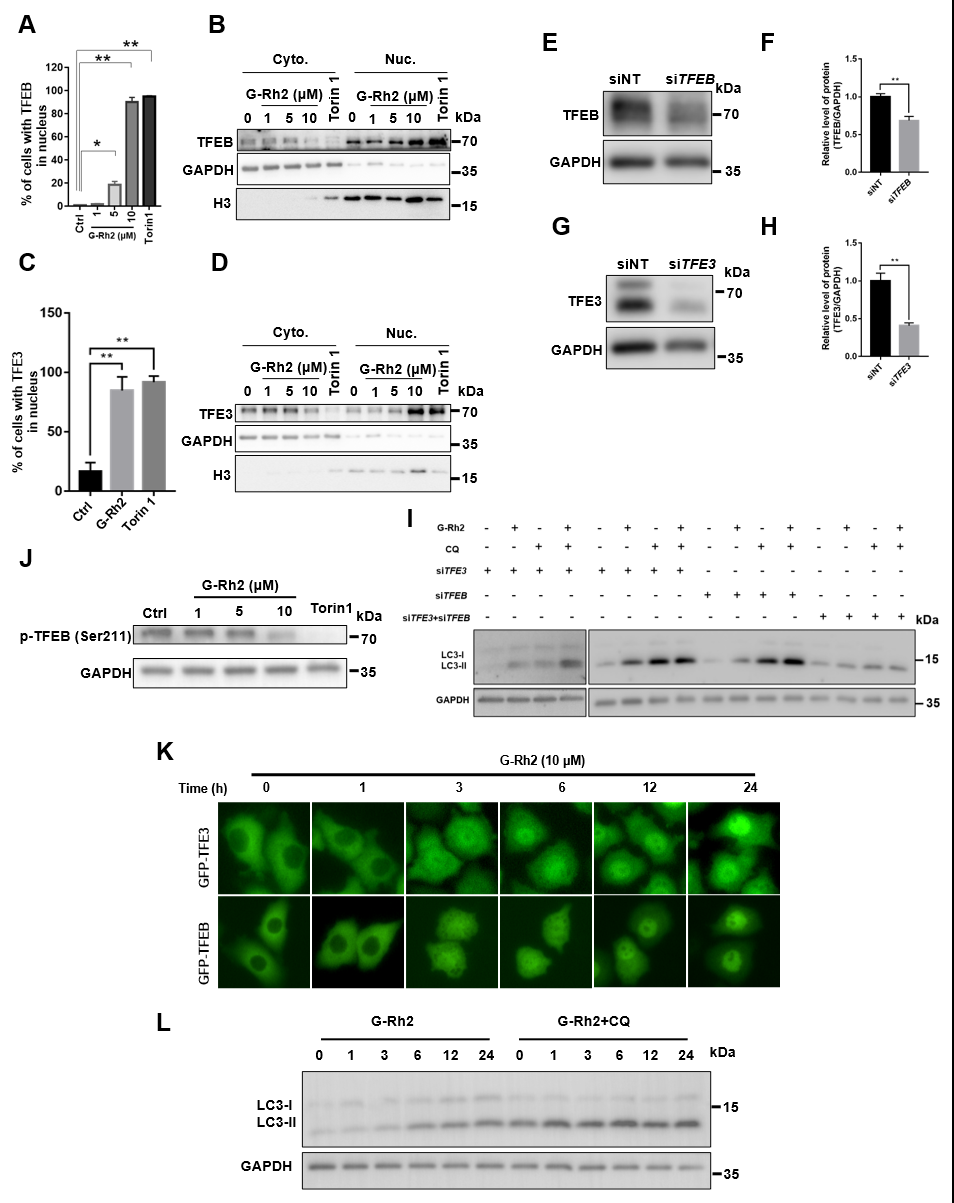


**Figure S1.** **Ginsenoside Rh2 induces autophagy via TFEB and TFE3.**

**(A)** G-Rh2 induces the relocation of TFEB from the cytoplasm into the nucleus. U2OS cells were incubated with G-Rh2 (1, 5, and 10 μM) for 16 h. Torin1(1 μM) treatment was used as a positive control. The subcellular distribution of TFEB in Figure 1H was quantified. Scale bar: 15 μm. **(B**) G-Rh2 promotes the nuclear accumulation of endogenous TFEB. U2OS cells were incubated with G-Rh2 (1, 5, and 10 μM) for 16 h. Torin1(1 μM) treatment was used as a positive control. The cytoplasm and nucleus TFEB contents were detected by western blotting. (**C**) G-Rh2 induces the relocation of TFE3 from the cytoplasm into the nucleus. U2OS cells transiently expressing GFP-N1-TFE3 were incubated with G-Rh2 (10 μM) for 16 h. Torin1(1 μM) treatment was used as a positive control. The subcellular distribution of TFE3 in Figure 1I was quantified. (**D**) G-Rh2 promotes the nuclear accumulation of endogenous TFE3. After the incubation of U2OS cells with G-Rh2 for 24h, the cytoplasm and nucleus levels of TFE3 were detected by western blotting. Torin1(1 μM) treatment was used as a positive control. (**E-I**) G-Rh2 promotes autophagy flux in TFEB/TFE3-dependent manner. U2OS cells were transfected with specific siRNA for both TFEB and TFE3 (I) for 48h, and the expression of TFEB and TFE3 was measured (E and G) and quantified (F and H). After the knockdown of TFE3, TFEB, or both TFE3 and TFEB, LC3-II level was detected to reflect the autophagic flux in U2OS cells (I). (**J**) G-Rh2 reduces phosphorylated TFEB at Ser211. (**K**) G-Rh2 promotes the nuclear accumulation of TFEB and TFE3 in a time-dependent manner. (**L**) G-Rh2 increases LC3-II levels in a time-dependent manner, and G-Rh2 further increases LC3-II levels in the presence of lysosomal inhibitor CQ. A well-known mTOR inhibitor Torin 1 was used as a positive control. *, p<0.05, **, p<0.01.


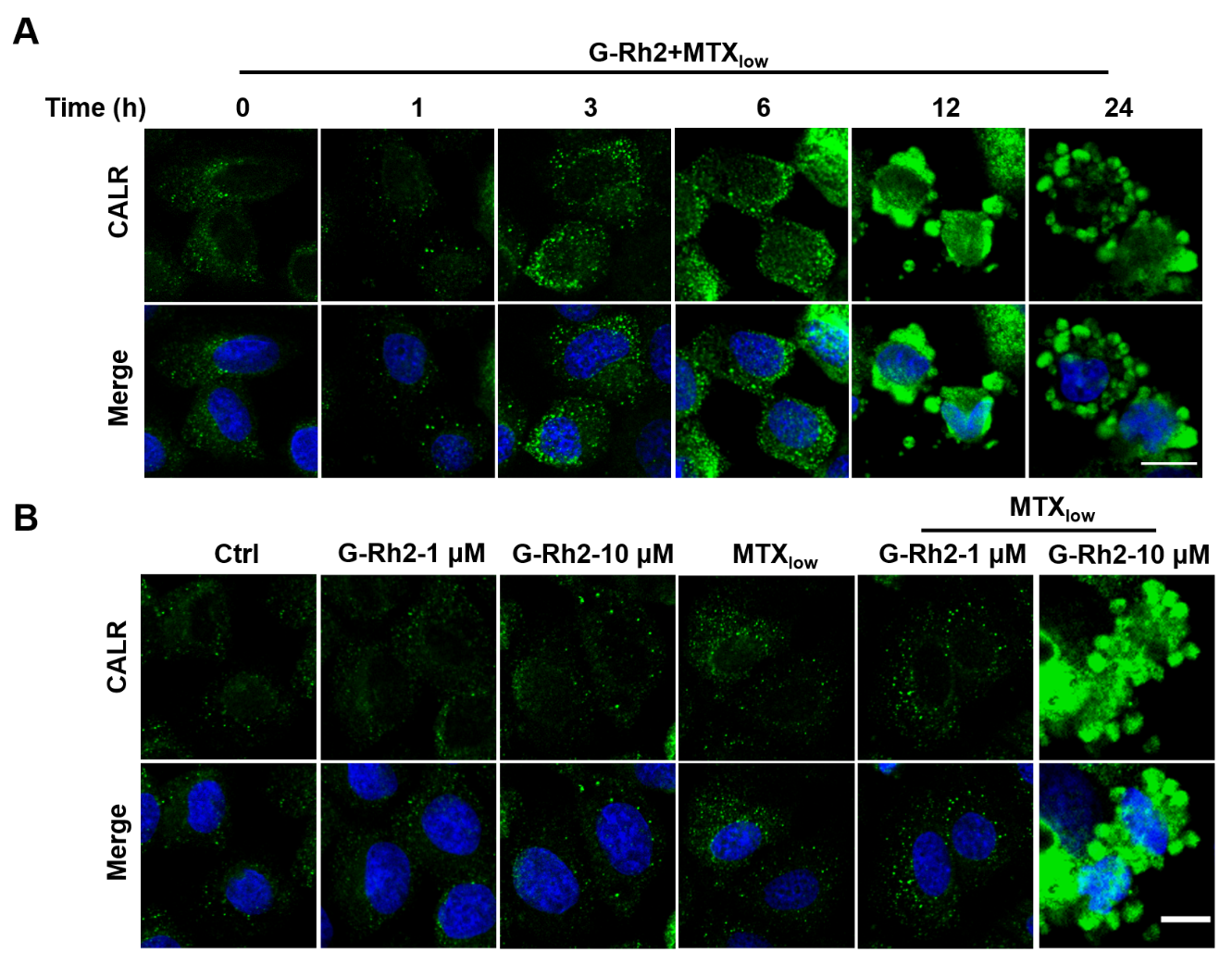


**Figure S2. Effects of Ginsenoside Rh2 plus MTX on ICD hallmarks induction.**

(**A**) G-Rh2 (10 μM) plus MTX_low_ (1 μM)-induced cell surface CALR exposure is in a time-dependent manner. After treatment of U2OS cells with G-Rh2 plus MTX_low_ in indicated time point (0-24 h), endogenous CALR was stained and recorded. (**B**) G-Rh2 (10 μM) plus MTX_low_ (1 μM)-induced cell surface CALR exposure is in a dose-dependent manner. After treatment of U2OS cells with indicated dose of G-Rh2 (1 μM or 10 μM), MTX_low_, or G-Rh2 (1 μM or 10 μM) plus MTX_low_ for 24 h, endogenous CALR was stained and recorded.


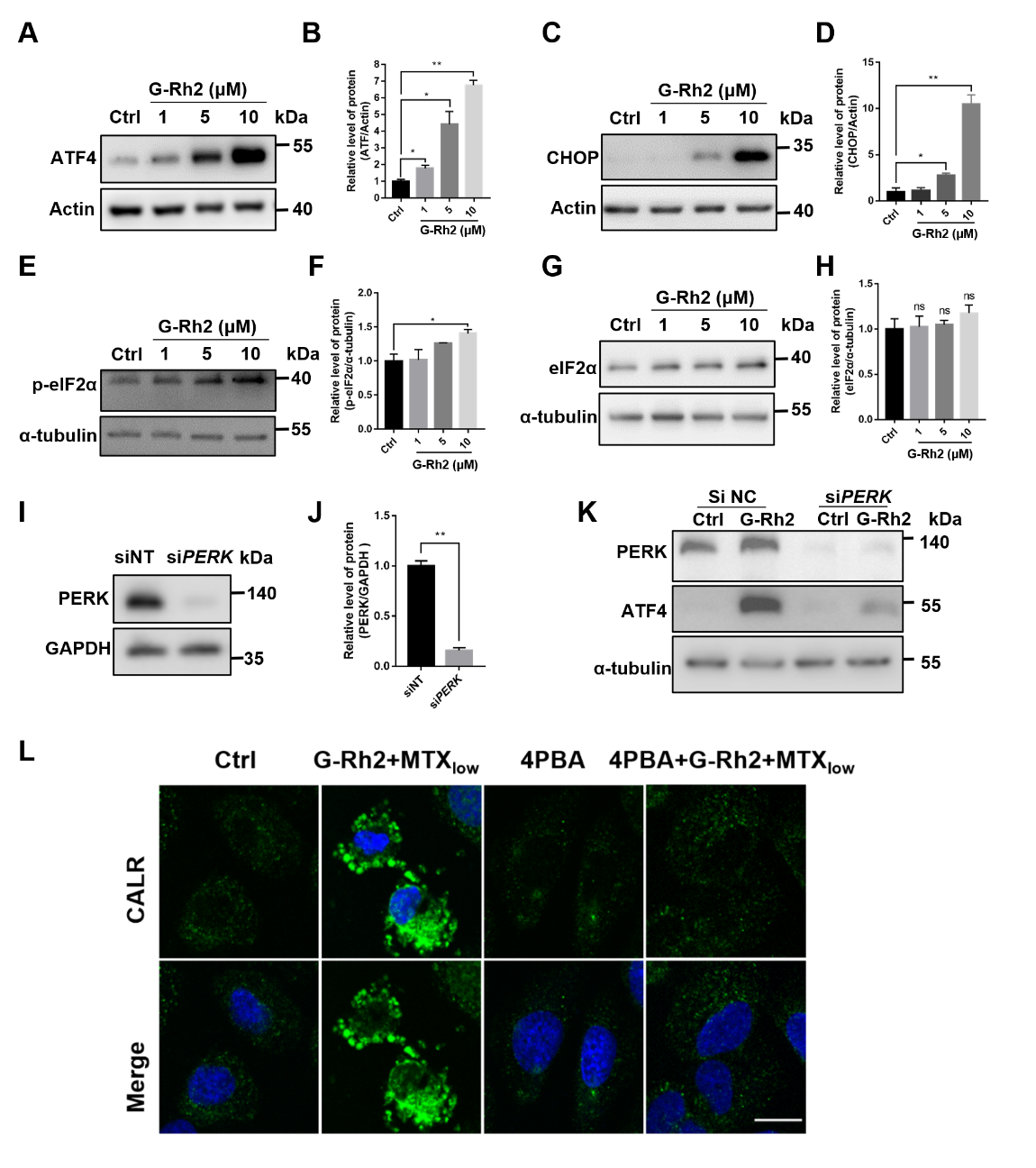


**Figure S3. Ginsenoside Rh2 enhances MTX-induced cell surface CALR exposure via the ER stress pathway.**

(**A** and **B**) G-Rh2 increases the expression of an ER stress marker ATF4. U2OS cells were incubated with G-Rh2 (1 μM, 5 μM, and 10 μM) for 16 h, and ATF4 levels were measured (**A**) and quantified (B). (**C** and **D**) G-Rh2 increases the expression of the ER stress marker CHOP. The expression of CHOP was detected after drug treatment as similarly in (A). (**E-H**) G-Rh2 increases p-eIF2α levels but not total eIF2α levels. The expression of p-eIF2α (E and F) and total eIF2α (G and H) were detected by western blotting and quantified after drug treatment. (**I** and **J**) U2OS were transfected with PERK-specific siRNA for 48 h and the expression of PERK was measured. (**K**) After *PERK* knockdown, ATF4 levels were detected after incubation with G-Rh2 for 24 h. (**L**) ER stress inhibitor 4-PBA (4-phenylbutyric acid) attenuates G-Rh2 plus MTX_low_ (1 μM)-induced cell surface CALR exposure. *, p<0.05; **, p<0.01.


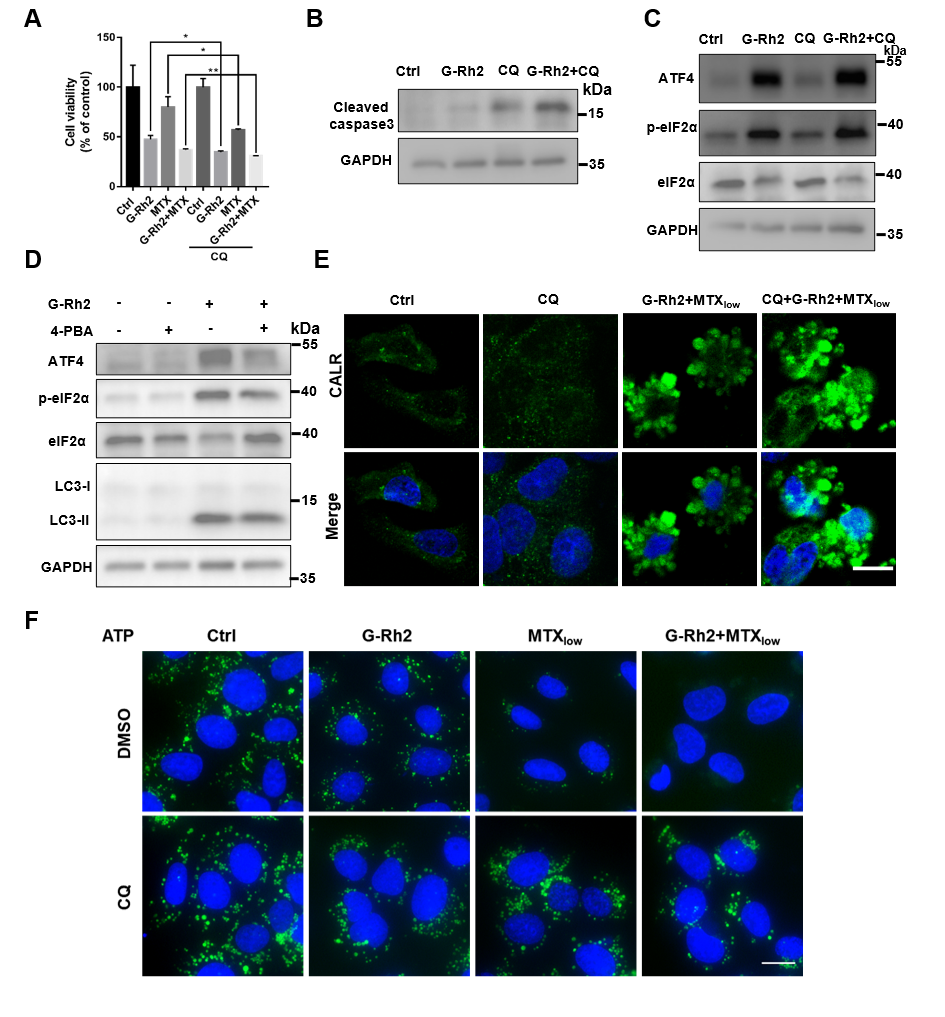


**Figure S4. Crosstalk between autophagy and ER stress pathway during Ginsenoside Rh2 plus MTX-induced ICD.**

(**A**) Inhibition of lysosomal functions with lysosomal inhibitor chloroquine (CQ) further increases G-Rh2 and MTX-induced apoptosis. U2OS cells were incubated with G-Rh2 (10 μM), MTX_low_ (1 μM) alone or in their combination in the presence or absence of CQ for 16 h, and cell viability was determined by CCK-8 assay. (**B**) Lysosomal inhibitor CQ further increases G-Rh2 (10 μM) plus MTX_low_ (1 μM)-induced apoptotic marker cleaved Caspase 3 levels. (**C**) Lysosomal inhibitor CQ does not further increase ER stress marker ATF4 and p-eIF2α levels. (**D**) ER stress inhibitor 4-PBA (4-phenylbutyric acid) attenuates G-Rh2-induced increase of autophagy marker LC3-II levels. (**E**) Lysosomal inhibitor CQ does not further increase G-Rh2 (10 μM) plus MTX_low_ (1 μM)-induced cell surface CALR exposure. (**F**) Lysosomal inhibitor CQ attenuates G-Rh2 (10 μM) plus MTX_low_ (1 μM)-induced intracellular ATP release as reflected by quinacrine staining.


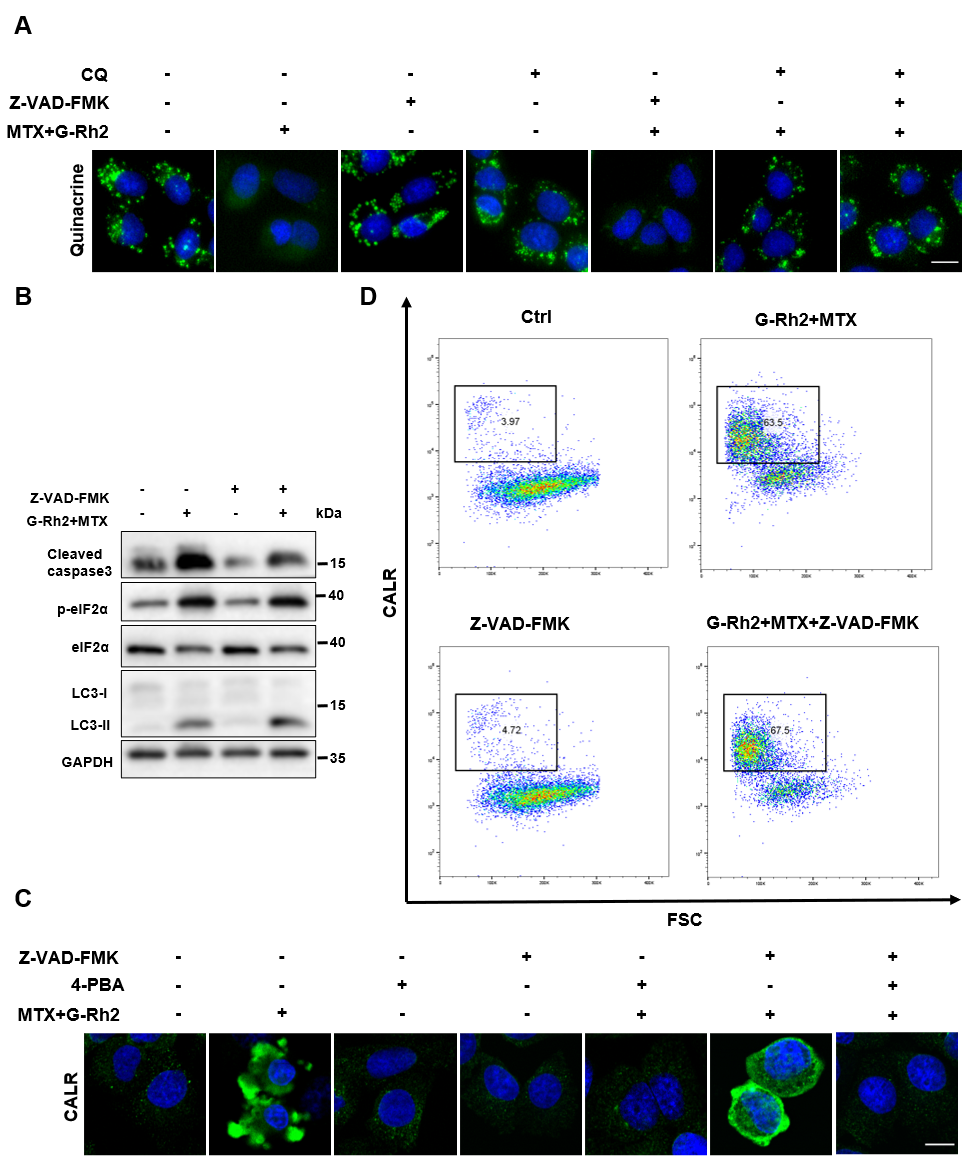


**Figure S5.** **Ginsenoside Rh2 plus MTX-induced ICD markers are independent of apoptosis.**

**(A)** Autophagy inhibitor CQ rather than apoptosis inhibitor Z-VAD-FMK inhibits G-Rh2 plus MTX-induced intracellular ATP release. U2OS cells were incubated with apoptosis inhibitor Z-VAD-FMK alone or in their combination in the presence or absence of G-Rh2 (10 μM) plus MTX_low_ (1 μM), intracellular ATP release was determined as reflected by quinacrine staining. **(B)** Apoptosis inhibitor Z-VAD-FMK did not inhibit G-Rh2 (10 μM) plus MTX_low_ (1 μM) ER stress marker p-eIF2α, and autophagy marker LC-II. U2OS cells were incubated with apoptosis inhibitor Z-VAD-FMK alone or in combination in the presence or absence of G-Rh2 (10 μM) plus MTX_low_ (1 μM), expression of an apoptosis marker cleaved Caspase 3, ER stress marker p-eIF2α, and autophagy marker LC3-II were detected by western blotting. **(C-D)** Apoptosis inhibitor Z-VAD-FMK did not inhibit G-Rh2 (10 μM) plus MTX_low_ (1 μM)-induced cell surface CALR exposure as reflected by immunostaining and flow cytometry assay.


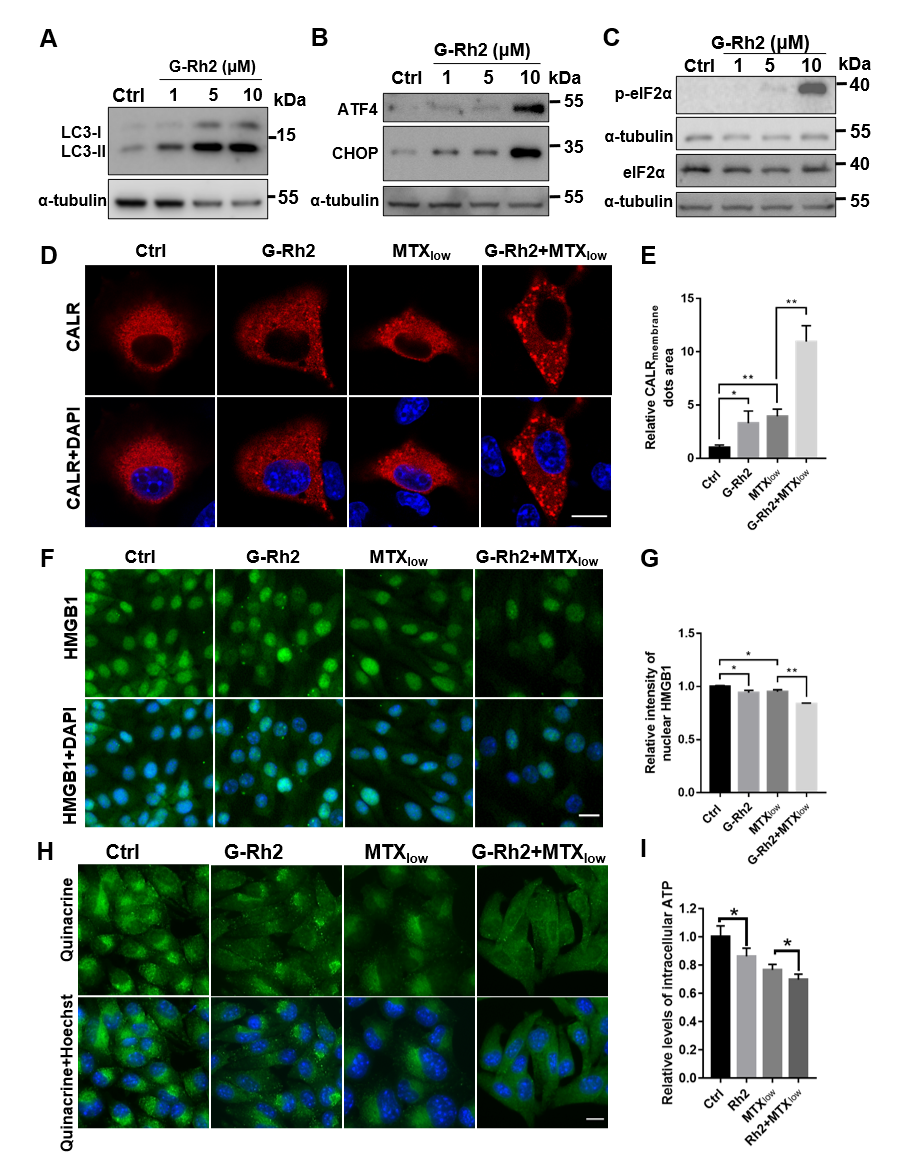


**Figure S6.** **Ginsenoside Rh2 induces autophagy and ER stress and promotes MTX-induced ICD in MCA205 fibrosarcoma cells.**

(**A**) G-Rh2 increases autophagy. MCA205 cells were incubated with G-Rh2 (1 μM, 5 μM, and 10 μM) for 16 h, and autophagic marker LC3-II levels was measured by western blotting. (**B** and **C**) G-Rh2 increases ER stress. MCA205 cells were treated with G-Rh2 (1 μM, 5 μM, and 10 μM) for 16 h, ER stress markers ATF4, CHOP (B), and p-eIF2α and eIF2α (C) levels were measured by western blotting. (**D** and **E)** G-Rh2 enhances MTX-induced cell surface CALR exposure. MCA205 cells transiently expressing CALR-KDEL-RFP were treated with a low concentration of MTX (MTX_low_) with or without G-Rh2 for 16 h, cell surface CALR exposure was visualized by a confocal microscope (**D**) and quantified (**E**). Scale bar: 15 μm. (**F** and **G**) G-Rh2 enhances MTX-induced HMGB1 release. HMGB1 were stained and visualized by a confocal microscope (**F**) and the nuclear HMGB1 contents were quantified (**G**) after drug treatment as shown in D. Scale bar: 15 μm. (**H** and **I**) G-Rh2 enhances MTX-induced ATP release. Intracellular ATP was detected by quinacrine staining (**H**) and quantified (**I**) after drug treatment as shown in D. Scale bar: 15 μm. *, p<0.05; **, p<0.01.


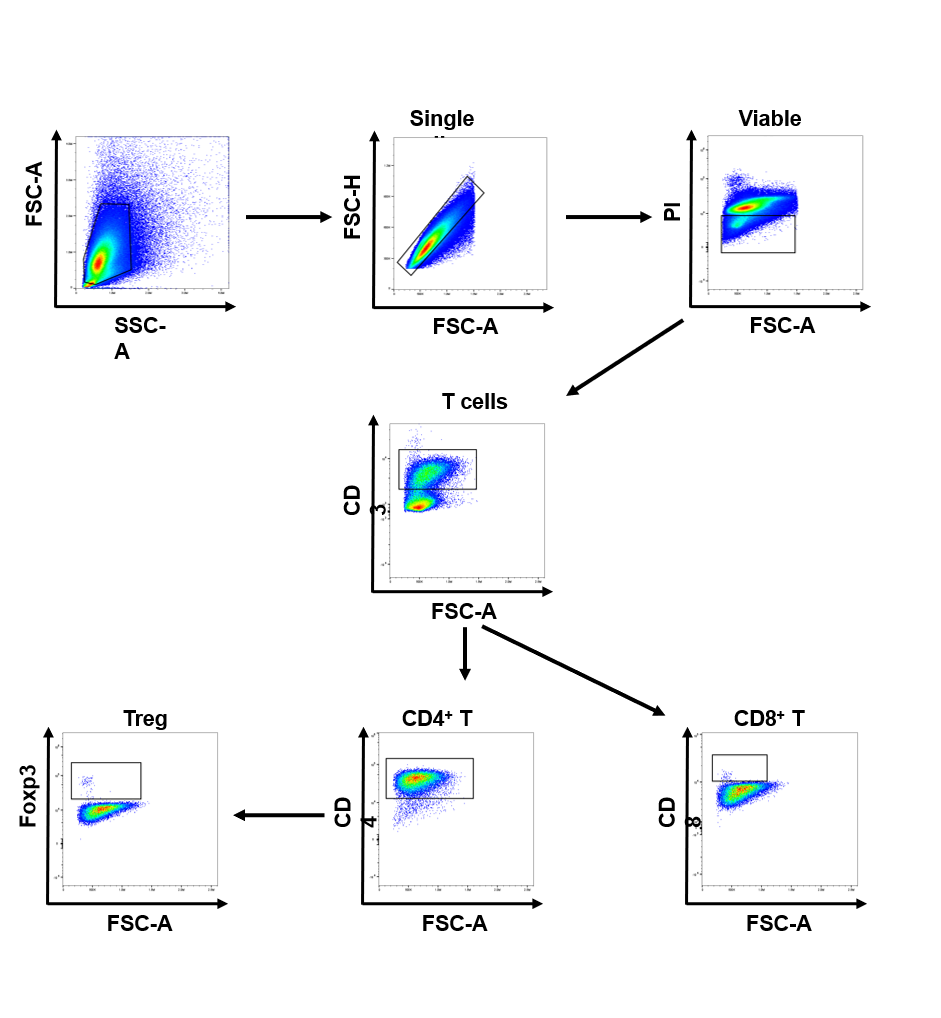


**Figure S7. Gating strategy for different cell populations in mice tumors by FACS**
