# Supplementary material for: A novel autophagy activator ginsenoside Rh2 enhances the efficacy of immunogenic chemotherapy
Source: Clin Transl Med. 2023 Feb 2;13(2):e1109. doi: 10.1002/ctm2.1109 (PMC9894730; doi:10.1002/ctm2.1109)
Supplement: Supplementary file 2 — Supporting Information [file CTM2-13-e1109-s001.docx]

**MATERIALS AND METHODS**

**Reagents and antibodies**

Ginsenoside Rh2 (A0241) was ordered from Chengdu Must Bio. MTX (S2485) and quinacrine dihydrochloride dihydrate (S5435) were purchased from Selleckchem. Chloroquine (C6628), anti-TFE3 antibody (HPA023881), anti-Flag antibody (F3165), and ATP Bioluminescence Assay Kit CLS II (11699695001) were purchased from Sigma. Torin 1 (2273-5) was purchased from BioVision. Hoechst (C1027) was purchased from Beyotime. BSA (A8020) was purchased from Solarbio. PBS (SH30256.01) was purchased from Hyclone. Trypsin-EDTA (25200114) and Opti-MEM (31985070) were ordered from Gibco. An anti-LC3 antibody (NB100-2220) was obtained from Novus Biologicals. Anti-GAPDH antibody (gtx100118) was purchased from GeneTex. An anti-TFEB antibody (303-673A) was purchased from Bethyl. Anti-Actin antibody (MA5-11869), anti-CD3e Monoclonal Antibody APC eBioscience™ (17-0031-82), anti-CD8a Monoclonal Antibody PE eBioscience™ (12-0081-82), anti-CD4 Monoclonal Antibody PerCP-Cyanine5.5 eBioscience™ (45-0042-82), anti-FOXP3 Monoclonal Antibody FITC eBioscience™ (11-5773-82), Goat anti-Mouse IgG (H+L) Secondary Antibody HRP (31430), Goat anti-Rabbit IgG (H+L) Secondary Antibody HRP (31460), Alexa Fluor®488 goat anti-Rabbit IgG (A-11034) and eBioscience™ Foxp3/ Transcription Factor Staining Buffer Set (00-5523-00) were purchased from Thermo Fisher Scientific. Anti-ATG5 antibody (12994), anti-Histone H3 antibody (9715S), anti-ATF4 antibody (11815S), anti-CHOP antibody (2895), anti-eIF2α antibody (9722), anti-PERK antibody (5683) and anti-Phospho-eIF2α (Ser51) antibody (9721S) were purchased from CST. Anti-HMGB1 antibody (ab18256) and anti-Calreticulin antibody (ab92516) were purchased from Abcam. Anti-Tubulin antibody (11224-1-AP) was purchased from Proteintech. In Vivo Mab anti-mouse CD16/CD32 (BE0307) was purchased from BioXcell. siRNA was purchased from Ribobio. Tumor Dissociation Kit (130-096-730) and Red Blood Cell Lysis Solution (Miltenyi Biotec 130-094-183) were purchased from Miltenyi Biotec.

**Cell culture**

U2OS cells and MCA205 cells purchased from ATCC were maintained in DMEM (C11995500BT), supplemented with 10% FBS, 100 U/mL penicillin, and 100 mg/mL streptomycin. For drug treatment, to reduce serum-mediated interference, the complete medium was changed by Opti-MEM with 1% FBS, and then treated with the indicated compounds.

**Cell transfection**

For overexpression experiments, tf-LC3, GFP-LC3, Flag-TFEB, GFP-TFE3, and CARL-KDEL-RFP plasmids ^1, 2, 3^ were transfected into cells via Lipofectamine 3000 (Thermo L3000015) reagent as described previously.^4^ To knock down specific genes including *PERK*, *TFEB*, *TFE3,* and *ATG5* as described previously^5^ in U2OS cells, lipofectamine™ RNAiMAX Transfection Reagent (Thermo 13778075) was used. Short interfering RNA (siRNA) sequences of relevant target genes were shown in Supplementary **Table S1**.

**Western blotting**

Cells were washed with PBS and lysed in RIPA lysis buffer (CST 9806). Cells were then collected by centrifugation (14, 000 g, 15 min). After quantification of protein concentration by using a BCA protein assay kit (Thermo 23225), 10-30 μg protein samples were heat-denatured (95℃, 5 min) before being loaded for protein separation using 10-15 % SDS-PAGE. Followed by gel electrotransferring, incubation with indicated primary and secondary antibodies blocking, relative protein bands were visualized by using SuperSignal™ West Femto Maximum Sensitivity Substrate (Thermo 34094) kit and captured by ChemiScope 6000 Touch (Clin X).

**Isolating** **proteins from cytoplasm and nucleus**

We isolated proteins from the cytoplasm and nucleus according to the previous protocols.^6, 7^ Three types of buffer were used to isolate the proteins from the cytoplasm and nucleus, including Buffer A (10 mM KCl, 1.5 mM MgCl_2_, 0.34 M sucrose, 1 mM dithiothreitol (DTT), 10% glycerin and 0.1% Triton X-100), Buffer B (10 mM KCl, 1.5 mM MgCl_2_, 0.34 M sucrose, 1 mM DTT), and Buffer C (3 mM EDTA, 0.2 mM EGTA and 1 mM DTT). All three buffers were supplemented with 1 mM PMSF. Briefly, cells per well were harvested and washed with PBS. Then cells were resuspended in buffer A on ice for 5 min and centrifuged at 1,500 g for 4 min at 4℃. The protein supernatant was harvested as the cytosolic extract. The pellets at the bottom were washed with Buffer B twice and centrifuged at 1,500 g for 4 min at 4℃. The pellets were then resuspended with Buffer C on ice for 10 min. Both cytosolic and nuclear extracts were stored at -80℃ until use.

**Immunofluorescence**

Cells were seeded on the coverslips which were placed in 24-well cell culture plates. After treatment with drugs, cells were fixed with 4% paraformaldehyde (PFA), permeabilized with 0.25% Triton X-100, and blocked with 2% BSA. Then cells were stained with indicated primary antibodies and then incubated with Alexa Fluor®488 (green) secondary antibody. Slides were visualized using a confocal laser scanning microscope (Leica TCS SP8 STED) or fluorescent microscope (Leica DFC7000 T). The data were analyzed using ImageJ software. For quantification of the TFEB/TFE3 accumulation was performed according to a previous protocol ^6, 8^. Briefly, images were acquired randomly from at least ten different fields per sample. Quantitation was done from each image for each sample, and around 100 cells per treatment from three independent experiments were performed to generate the graphed values.

**ATP release assays**

For intracellular ATP detection, cells were stained with 5 μM quinacrine ^3, 9^ for 10 min at 37℃. Meanwhile, the nucleus was stained with Hoechst. ATP staining was observed and captured by a fluorescent microscope (Leica DFC7000 T). The data were analyzed using ImageJ software.

Cell culture medium was collected for extracellular ATP detection after drug treatment. The medium/standard samples and luciferase reagent were added in a ratio of 1:1. The results were acquired using a multimode microplate reader (SPARK 10M) and analyzed by GraphPad Prism.

**Flow cytometry analysis**

To detect CALR exposure on the surface, U2OS cells were harvested, washed, and then resuspended in FACS buffer (1% BSA in PBS) containing anti-CARL antibody (1:50 dilution) and incubated on ice for 30 min^3^. Cells were then fixed with 4% PFA and followed by incubation with Alexa Fluor®488 (green) secondary antibody for 30 min on ice before being resuspended with 200 μL FACS buffer for washing. Cells were finally resuspended in PBS for Flow Cytometry analysis.

Tumor immune infiltration detection was performed according to previous protocol. Briefly, tumor tissues were harvested, weighed, and cut into small pieces before being transferred into gentle MACS C tube for enzyme digestion and dissociation. After dissociation, homogenates were filtered by a 70 μm cell strainer and centrifuged to discard the supernatant. Then the erythrocytes of homogenates were removed by a Red Blood Cell Lysis Solution kit and washed with PBS 3 times. The viability of homogenates resuspended in PBS was measured and 1x10^6^ cells were collected for flow cytometry analysis. Cells were stained with PI and anti-CD16/CD32, anti-CD3e, anti-CD4, and anti-CD8a antibodies. After fixation and permeabilization with eBioscience™ Foxp3/Transcription Factor Staining Buffer Set, the cells were stained with an anti-FOXP3 antibody. Finally, cells were resuspended in PBS for flow cytometry analysis (Cytek® NL-CLC). Data were analyzed by Flowjo 10.

***In vivo* experiment**

Male C57BL/6 mice (6-8 weeks old) were fed in a Specific Pathogen Free (SPF) house with a regular 12-hour light/dark cycle, nutritious food, and sterilized water. After one-week adaption to the environment, mice were inoculated with 5x10^5^ MCA205 cells according to previous studies.^10^ When tumors appeared palpable (around 7 days), mice were intraperitoneally injected with G-Rh2 (30 mg/kg). One and two days later, MTX (5.17mg/kg) and G-Rh2 (30 mg/kg) were injected into mice, respectively. On the following days, G-Rh2 (30 mg/kg) was given three times per week. Tumor growth and mice weight were documented every two days. Mice were sacrificed at the endpoint or obvious discomfort signs were observed.

**Statistical analysis**

For comparison between the two groups, data were analyzed by Student’s *t*-test. The data were presented as mean ± SEM from at least three independent trials using GraphPad Prism 7.0. Significant differences were claimed as *p < 0.05 and **p < 0.01.

**Supplementary Table 1**

**Table S1. siRNA sequences**

| siRNA | Target Sequence |
| --- | --- |
| siTFEB | CUACAUCAAUCCUGAAAUG |
| siTFE3 | GGAAUCUGCUUGAUGUGUA |
| siPERK | GGAACGACCTGAAGCTATA |
| siATG5 | CAACTTGTTTCACGCTATA |

**References:**

1. Menger, L. *et al.* Cardiac glycosides exert anticancer effects by inducing immunogenic cell death. *Science translational medicine* **4**, 143ra199 (2012).

2. Obeid, M. *et al.* Calreticulin exposure dictates the immunogenicity of cancer cell death. *Nature medicine* **13**, 54-61 (2007).

3. Galluzzi, L. *et al.* Consensus guidelines for the definition, detection and interpretation of immunogenic cell death. *Journal for immunotherapy of cancer* **8** (2020).

4. Yang, C.B. *et al.* TFEB, a master regulator of autophagy and biogenesis, unexpectedly promotes apoptosis in response to the cyclopentenone prostaglandin 15d-PGJ2. *Acta pharmacologica Sinica* **43**, 1251-1263 (2022).

5. Chen, G. *et al.* 3,4-Dimethoxychalcone induces autophagy through activation of the transcription factors TFE3 and TFEB. *EMBO molecular medicine* **11**, e10469 (2019).

6. Yang, C. *et al.* A stress response p38 MAP kinase inhibitor SB202190 promoted TFEB/TFE3-dependent autophagy and lysosomal biogenesis independent of p38. *Redox biology* **32**, 101445 (2020).

7. Weinberg, D.N. *et al.* The histone mark H3K36me2 recruits DNMT3A and shapes the intergenic DNA methylation landscape. *Nature* **573**, 281-286 (2019).

8. Medina, D.L. *et al.* Lysosomal calcium signalling regulates autophagy through calcineurin and ​TFEB. *Nature cell biology* **17**, 288-299 (2015).

9. Forveille, S. *et al.* Quinacrine-mediated detection of intracellular ATP. *Methods in enzymology* **629**, 103-113 (2019).

10. Wu, Q. *et al.* Isobacachalcone induces autophagy and improves the outcome of immunogenic chemotherapy. *Cell death & disease* **11**, 1015 (2020).
